# Supplementary material for: C-reactive protein: An easy marker for early differentiation between leptospirosis and dengue fever in endemic area
Source: PLoS One. 2023 May 17;18(5):e0285900. doi: 10.1371/journal.pone.0285900 (PMC10191341; doi:10.1371/journal.pone.0285900)
Supplement: S1 Table — *Regarding symptoms and other physical signs not reported, if a symptom/sign was not mentioned during the retrospective review of the medical charts then it was considered as absent. Therefore, there is no data considered as missing data for the symptom category. ALP: alkaline phosphatase; aPTT: activated partial thromboplastin time; AST: aspartate aminotransferase; ALT: alanine aminotransferase; BUN: blood urea nitrogen; CK: creatinine kinase; CRP: C-reactive protein; DBP: diastolic blood pressure; GGT: gamma-glutamyl transferase; RBC: red blood cell; SBP: systolic blood pressure. (DOCX) [file pone.0285900.s001.docx]

S1 Table: Comparison of clinical and biological factors at initial hospital presentation of leptospirosis and dengue fever cases on Reunion Island between 2018 and 2019 (bivariate analysis)

| Variable* | Leptospirosis  (N=98) | Missing data, N | | | Dengue fever  (N=673) | Missing data, N | | | P-value | |
| --- | --- | --- | --- | --- | --- | --- | --- | --- | --- | --- |
| Socio-demographic data | | | | | | | | | | |
| Age, years, mean(±SD) | 47.8 (±17.1) | 0 | | | 48.9 (±23.3) | 0 | | | 0.519 | |
| Sex, Male N (%) | 89 (91%) | 0 | | | 299 (44%) | 0 | | | **<0.001** | |
| Time before admission, days, mean(±SD) | 3.9 (±2.1) | 1 | | | 2.5 (±2.5) | 16 | | | **<0.001** | |
| Physical examination |  |  | | |  |  | | |  | |
| Fever >38.5°C, N (%) | 25 (26%) | 1 | | | 242 (38%) | 28 | | | **0.022** | |
| Pulse rate, per min, mean(±SD) | 101 (±19) | 0 | | | 96 (±37) | 27 | | | 0.186 | |
| DBP, mmHg, mean(±SD) | 70 (±14) | 1 | | | 73 (±13) | 41 | | | **0.024** | |
| SBP, mmHg, mean(±SD) | 122 (±22) | 1 | | | 127 (±23) | 41 | | | **0.030** | |
| Biological features,  mean(±SD) |  |  | | |  |  | | |  | |
| Hemoglobin, g/dL | 13.3 (±1.8) | 1 | | | 13.4 (±2.0) | 3 | | | 0.581 | |
| Hematocrit, % | 37.9 (±5.0) | 2 | | | 39.3 (±5.2) | 3 | | | **0.010** | |
| RBC mean corpuscular volume, fL | 83 (±9) | 3 | | | 85 (±6) | 3 | | | 0.101 | |
| Leukocytes count, ×10^9^/L | 10.8 (±4.0) | 1 | | | 5.1 (±3.3) | 3 | | | **<0.001** | |
| Neutrophils count, ×10^9^/L | 9.3 (±3.8) | 1 | | | 3.5 (±2.6) | 19 | | | **<0.001** | |
| Lymphocytes count, ×10^9^/L | 0.80 (±0.49) | 2 | | | 0.86 (±1.44) | 19 | | | 0.679 | |
| Monocytes count, ×10^9^/L | 0.67 (±0.38) | 2 | | | 0.56 (±0.34) | 19 | | | **0.003** | |
| Platelets count, ×10^9^/L | 103 (±72) | 1 | | | 165 (±79) | 3 | | | **<0.001** | |
| aPTT ratio over control value | 1.10 (±0.13) | 28 | | | 1.18 (±0.22) | 126 | | | **0.005** | |
| Plasma creatinine, µmol/L | 278 (±268) | 2 | | | 106 (±108) | 26 | | | **<0.001** | |
| BUN, mmol/L | 13.8 (±11.1) | 3 | | | 5.6 (±4.7) | 21 | | | **<0.001** | |
| AST, IU/L | 104 (±104) | | 4 | 101 (±541) | | | 58 | 0.957 | |  |
| ALT, IU/L | 69 (±56) | | 5 | 60 (±225) | | | 62 | 0.707 | |  |
| ALP, IU/L | 94 (±34) | | 16 | 83 (±54) | | | 251 | 0.082 | |  |
| GGT, IU/L | 86 (±73) | | 26 | 65 (±144) | | | 213 | 0.230 | |  |
| Total bilirubin, µmol/L | 84.5 (±103.6) | | 11 | 10.9 (±33.7) | | | 239 | **<0.001** | |  |
| Serum sodium, mmol/L | 133.3 (±4.4) | | 2 | 136.5 (±3.3) | | | 21 | **<0.001** | |  |
| Serum potassium, mmol/L | 3.7 (±0.5) | | 1 | 3.9 (±0.5) | | | 78 | **<0.001** | |  |
| Serum chlorine, mmol/L | 93.2 (±5.5) | | 3 | 98.9 (±4.4) | | | 32 | **<0.001** | |  |
| Serum calcium, mmol/L | 2.21 (±0.13) | | 37 | 2.26 (±0.12) | | | 150 | **0.002** | |  |
| CK, IU/L | 2144 (±3176) | | 17 | 607 (±7145) | | | 134 | **<0.001** | |  |
| CRP, mg/L | 229.2 (±122.1) | | 0 | 18.5 (±30.3) | | | 40 | **<0.001** | |  |

*Regarding symptoms and other physical factors not reported, if a symptom/sign was not mentioned during the retrospective review of the medical charts then it was considered as absent. Therefore, there is no data considered as missing data for the symptom category.

ALP: alkaline phosphatase; aPTT: activated partial thromboplastin time; AST: aspartate aminotransferase; ALT: alanine aminotransferase; BUN: blood urea nitrogen; CK: creatinine kinase; CRP: C-reactive protein; DBP: diastolic blood pressure; GGT: gamma-glutamyl transferase; RBC: red blood cell; SBP: systolic blood pressure.
