# Supplementary material for: C-reactive protein: An easy marker for early differentiation between leptospirosis and dengue fever in endemic area
Source: PLoS One. 2023 May 17;18(5):e0285900. doi: 10.1371/journal.pone.0285900 (PMC10191341; doi:10.1371/journal.pone.0285900)
Supplement: S2 Table — ICU: intensive care unit. (DOCX) [file pone.0285900.s002.docx]

S2 Table: Main outcomes of confirmed cases of leptospirosis and dengue fever on Reunion Island during 2018 and 2019

| Outcomes | Leptospirosis  (N=98) | Dengue fever  (N=673) |
| --- | --- | --- |
| Hospitalization, N(%) | 89 (91) | 320 (48) |
| Length of stay, days, mean(range) | 7.6 (1-49) | 5.0 (1-30) |
| ICU among hospitalized, N(%) | 46 (52) | 49 (15) |
| Death, N(%) | 3 (3) | 12 (2) |

ICU: intensive care unit
