# Supplementary material for: C-reactive protein: An easy marker for early differentiation between leptospirosis and dengue fever in endemic area
Source: PLoS One. 2023 May 17;18(5):e0285900. doi: 10.1371/journal.pone.0285900 (PMC10191341; doi:10.1371/journal.pone.0285900)
Supplement: S3 Table — ICU: intensive care unit. (DOCX) [file pone.0285900.s003.docx]

**S3 Table:** Comparison of DF patients characteristics according to CRP threshold of 50mg/L on Reunion Island during 2018 and 2019

| Characteristics | CRP≤50mg/L  (N=592) | CRP>50mg/L  (N=41) | P-value |
| --- | --- | --- | --- |
| Age, years, mean(±SD) | 48.1 (±23.4) | 67.6 (±16.9) | **<0.001** |
| Background, N(%) |  |  |  |
| Diabetes | 107 (18) | 15 (37) | **0.007** |
| Hypertension | 172 (29) | 23 (56) | **0.001** |
| Chronic kidney disease | 44 (7) | 8 (20) | **0.014** |
| Hospitalization, N(%) | 269 (45) | 30 (73) | **<0.001** |
| Length of stay, mean(range) | 4.9 (1-30) | 6.6 (1-17) | **0.026** |
| ICU among hospitalized, N(%) | 33 (12) | 9 (30) | **0.022** |
| Severe dengue, N(%) | 123 (21) | 18 (44) | **0.001** |
| Coinfections, N(%) | 44 (7) | 17 (42) | **<0.001** |
| Death, N(%) | 8 (1) | 2 (5) | 0.132 |

ICU: intensive care unit; SD: standard deviation
