# Supplementary material for: C-reactive protein: An easy marker for early differentiation between leptospirosis and dengue fever in endemic area
Source: PLoS One. 2023 May 17;18(5):e0285900. doi: 10.1371/journal.pone.0285900 (PMC10191341; doi:10.1371/journal.pone.0285900)
Supplement: S4 Table — aPTT ratio: ratio of activated partial thromboplastin time of patient over control; AST: aspartate transaminase; CK: creatinine kinase; ESR: erythrocyte sedimentation rate; LR-: negative likelihood ratio, LR+; positive likelihood ratio, ND; not determined, MAT; microscopic agglutination test; NS1Ag: non-structural 1 antigen; PCR: polymerase chain reaction; Se: sensitivity; Sp: specificity; WBC: white blood cells. *Values are inferred from sensitivity and specificity from the studies according to the formulae: LR+ = Se/(1-Sp) and LR- = (1-Se)/Sp. (DOCX) [file pone.0285900.s004.docx]

**S4 Table:** Main studies reporting differentiating factors between leptospirosis and DF in multivariate analysis

| **Study** | **Libraty et al. [9]** | **Varma et al. [16]** | **Rajapakse et al. [17]** | **Le Turnier et al. [7]** | **Present study (Maillard et al)** |
| --- | --- | --- | --- | --- | --- |
| **Design** | Prospective | Prospective | Prospective | Retrospective | Retrospective |
| **Setting** | Hospital-based | ND | Hospital-based | Hospital-based | Hospital-based |
| **Area** | Thailand | India | Sri Lanka | French Guiana | Reunion island |
| **Study period** | 1994-1999 | 2006-2008 | 2012-2014 | 2007-2014 | 2018-2019 |
| **Sample size**  **(leptospirosis vs. DF)** | 18 vs. 214 | 100 vs. 100 | 232 vs. 175 | 72 vs. 216 | 98 vs 633 for CRP alone |
| **Population** | Children | Adults | Adults and children >12 | Adults and children >15 | Adults and children |
| **Case definition of leptospirosis** | Serology, MAT | Serology | MAT | PCR or serology | PCR or serology |
| **Case definition of DF** | PCR or serology | Serology | ND | NS1 Ag detection | PCR |
| **Main factors associated to leptospirosis in multiple regression model** | -neutrophil count increase of 1x10^9^/L  -plasma albumin decrease >10 g/L  -AST ranged >30 and <80 IU/mL | Association of:  -WBC >11x10^9^/L  -creatininemia >176 µmol/L  -bilirubin >34 µmol/L  -CK >500 UI/L  -albuminemia <30 g/L  -ESR >40mm | Composite scoring system:  -exposure  -neutrophil >80%  -platelets <85x10^9^/L  -creatininemia >150 µmol/L  -bilirubin >30 µmol/L | -WBC >10x10^9^/L  -bilirubin >20 µmol/L  -CRP >50mg/l | -Neutrophils  -Platelets  -aPTT ratio  -CRP |
| **Diagnosis performance** | If all criteria:  Se 83%  Sp 90%  LR+8.3^*^  LR- 0.19^*^ | If all criteria:  Se 98%  Sp 95%  LR+19.6^*^  LR-0.02^*^ | If score > threshold:  Se 80%  Sp 60%  LR+2.01  LR-0.32 | For CRP >50 mg/L:  Se 88.9%  Sp 95.2%  LR+18.5^*^  LR-0.12^*^ | For CRP >50 mg/L:  Se 93.9%  Sp 93.5%  LR+14.5  LR-0.07 |

aPTT ratio: ratio of activated partial thromboplastin time of patient over control; AST: aspartate transaminase; CK: creatinine kinase; ESR: erythrocyte sedimentation rate; LR-: negative likelihood ratio, LR+; positive likelihood ratio, ND; not determined, MAT; microscopic agglutination test; NS1Ag: non-structural 1 antigen; PCR: polymerase chain reaction; Se: sensitivity; Sp: specificity; WBC: white blood cells.

*Values are inferred from sensitivity and specificity from the studies according to the formulae: LR+ = Se/(1-Sp) and LR- = (1-Se)/Sp.
